# Microorganisms Linked to Health Care–Associated Infections: Modernization of Terminology Resources for Reporting to the National Healthcare Safety Network

**DOI:** 10.2196/66088

**Published:** 2025-08-19

**Authors:** Sheila Abner, Shuai Zheng, Caroline Macumber, Shaun Shakib, Jonathan Edwards, Andrea Benin

**Affiliations:** 1 Division of Healthcare Quality Promotion, National Center for Emerging and Zoonotic Infectious Diseases, Centers for Disease Control and Prevention, 1600 Clifton Road, Atlanta, GA, 30329, United States, 1 4047183262; 2 Clinical Architecture, Carmel, IN, United States

**Keywords:** SNOMED CT, terminology, microorganisms, health care–associated infections, public health surveillance

## Abstract

The National Healthcare Safety Network (NHSN) of the Centers for Disease Control and Prevention (CDC) needed a modernized approach to manage resources containing standardized terminology that specify microorganism data submitted electronically for legacy reporting. Health care–associated infections (HAIs) reported to NHSN require the submission of data regarding specific microorganisms attributed to the patient’s condition. Data on microorganisms submitted to the NHSN electronically must use the SNOMED CT terminology standard. Terminology artifacts that guide submission of microorganism data have been maintained in spreadsheets that have become increasingly challenging to manage. This case report details the initial use case for the implementation of off-the-shelf software within the NHSN to modernize the maintenance of terminology assets. Resources that guide reporting microorganisms for HAIs were used as a prototype to demonstrate how a software application can be practically implemented to streamline the maintenance of complex terminology assets. Mission-critical artifacts have been reconciled and consolidated into a single source of truth knowledgebase using an off-the-shelf software solution. This report shares progress and lessons learned regarding the modernization of NHSN’s *Pathogen Codes* resource and its derivative artifacts. A model is now available that can be replicated across other NHSN legacy artifacts. Our experience can be applied to other public health use cases and information systems facing similar challenges with attachments to legacy terminology resources and systems.

## Introduction

The National Healthcare Safety Network (NHSN) of the Centers for Disease Control and Prevention (CDC) is the nation’s surveillance program for health care–associated infections (HAIs) and other conditions, patient safety events, and antimicrobial use and resistance [1]. The NHSN collects and processes surveillance data for the purpose of promoting efforts to prevent adverse events in health care by enabling facilities and governance agencies to determine problematic areas and formulate corresponding corrective actions.

The NHSN was launched in 2005 as a web-based application, succeeding the smaller DOS-based National Nosocomial Infection Surveillance (NNIS) system that had been in operation since the 1980s [2]. A bellwether for the adoption of HL7 data standards and electronic public health reporting, the NHSN implemented its first clinical document architecture (CDA) data submissions in 2008. As of November 2024, there were more than 165,000 users and over 38,500 health care facilities across the health care continuum reporting to the NHSN. More than 59 CDA vendors have established the capacity to submit data electronically on behalf of facilities.

When the NHSN first enabled electronic reporting, specifications for value sets from standardized vocabularies were needed to guide CDA vendors. Spreadsheets became the de facto solution for managing those resources because terminology software was not available. Additional attributes of metadata and relationships to other artifacts were incorporated into the spreadsheets to meet business needs. Maintaining these assets became labor-intensive with the growth of the NHSN expanding beyond its original functions.

As part of the CDC’s Data Modernization Initiative, the NHSN has adopted a new software platform for the maintenance of terminology assets. This paper highlights the NHSN’s early successes in modernizing resources that guide standardized data collection of microorganisms attributed to HAIs while preserving backward compatibility with legacy business processes.

## Evaluation of Legacy State and Transition Approach

Terminology resource management processes and products were qualitatively evaluated before and after the adoption of an off-the-shelf solution. An assessment of the historical state was performed by terminologists and informaticists skilled in the laboratory data domain. Stakeholders—including data scientists, database administrators, system developers, in addition to epidemiologists and infection preventionists authoring surveillance protocols—were questioned in focus groups to determine pitfalls and pain points in legacy processes. Feedback and insight from those interviews guided the overarching business and technical requirements for the transition activities.

The deployment of the new platform has been approached with three major features. First, the required servers have been constructed in the cloud environment of the CDC, followed by integration and performance testing with other infrastructural systems and existing NHSN applications. Various data provisioning methods have also been explored and merged into the roadmap of the NHSN.

Second, a corresponding standard operating procedure (SOP) has been developed to guide usage of the platform and facilitate communication among collaborators. A Terminology Committee, including physician informaticists, has been assembled to centralize discussions of terminology-related topics and requests, providing convenient consultation services to all stakeholders. After requests are conceptualized, they are formally itemized into specific requirements and documented in standardized terminology asset management (TAM) request forms. The SOP improves the efficiency of communication and streamlines coauthoring and maintenance of terminology resources into the new platform.

Third, necessary training sessions have been scheduled for the software users and stakeholders. In addition to the basic operations of the software platform, fundamental informatics and terminology principles have been taught in the form of training videos, webinars, and workshops to educate the workforce of NHSN. Subgroups have also been defined to offer additional domain-specific trainings based on different roles in the workflow.

## Historical State and Drivers for Implementing Improvements

The NHSN has developed an extensive collection of terminology assets, including 2 code systems and more than 80 value sets from various reference terminologies for electronic data specifications conformant to the HL7 CDA standard [3]. In addition to value sets in the value set authority center (VSAC), a publicly available authoring and provisioning tool hosted by the National Library of Medicine [4], CDA vendors are also dependent on the NHSN information data model (IDM), which combines business rules with standardized terminology content. The IDM has been maintained in spreadsheets that evolved into a multi-faceted resource and was well-positioned for modernization. The first proof of concept for such modernization focused on a high-priority table in the IDM, *Pathogen Codes* [5], that catalogs criteria for reporting microorganisms linked to all HAI events in the existing NHSN surveillance portfolio.

The NHSN retained approximately 1000 microorganism terms from its NNIS predecessor and established a methodology to create unique and human readable codes for analysis purposes. These short representations for pathogen concepts were challenging to curate after being coupled to SNOMED CT [6] concept identifiers required for the electronic reporting option. Although the naming conventions for NHSN codes became less intuitive as the number of terms increased to keep up with growth of NHSN over the years, these conventions remained mandatory for analyzing data.

By 2019, the *Pathogen Codes* resource had amassed more than 3500 rows of terms that included subsets of synonyms. Synonyms were particularly challenging to manage due to the ever-changing landscape of microbial nomenclature [7]. More than 35 columns of metadata were needed to characterize the reporting criteria across a growing number of complex HAI events. The sheer number of mixed-term types with manually curated mnemonic codes and business logic attributes managed in spreadsheets led to the need for comprehensive terminology software for the most effective maintenance.

Although the SNOMED CT U.S. Edition releases occur biannually (March and September), updates to the *Pathogen Codes* resource have coincided with annual NHSN application major releases in January. Because the NHSN development life cycles also need to accommodate the development cycles of CDA vendors, the offset timing of NHSN releases and SNOMED CT updates meant that it was challenging to maintain up-to-date SNOMED CT content in distributed NHSN resources.

Updating the *Pathogen Codes* resource was so labor-intensive that the task needed to be started on a version of SNOMED CT that preceded the NHSN application release by more than a year. The terminologist prepared enumerated lists of SNOMED CT concepts in VSAC, and exported files were manually compared to existing *Pathogen Codes* content to identify inactivated codes, name changes to term descriptions, and additions being considered. The resulting marked-up spreadsheet that highlighted proposed updates was shared with protocols and data analysis stakeholders to reach consensus on changes to microorganism membership and associated attributes. Following that reconciliation, the annotated spreadsheet was submitted to business analysts with enough time for the development team to implement the changes for the January NHSN application releases. Overall, the maintenance process relied heavily on manually processed spreadsheets and email communication which intensified the workload and prolonged the path to completion. The fragmented process caused challenges to version control and rigorous traceability.

In addition, supplemental spreadsheets derived from the master *Pathogen Codes* table were required to support the divergent needs of the two different approaches available for data submission. Subsets of content needed to be parsed and simplified in a companion spreadsheet for users entering one event at a time into the web application. In contrast, almost 1000 additional terms needed to be curated separately to expand a subset of content (eg, more than 700 *E. coli* serotypes in SNOMED CT) to support users submitting data via CDA files.

## Evolution of Technology Advancement

Migrating the *Pathogen Codes* resource from a spreadsheet to an off-the-shelf software product has modernized how NHSN is maintaining and provisioning this critical terminology asset. The SNOMED CT code system now serves as the core structure for *Pathogen Codes*, and the standard content is enriched with NHSN-specific metadata. Key principles relevant to this effort are derived from the *Desiderata for Controlled Medical Vocabularies* that have influenced the design of several modern code systems [8].

**Figure 1.**
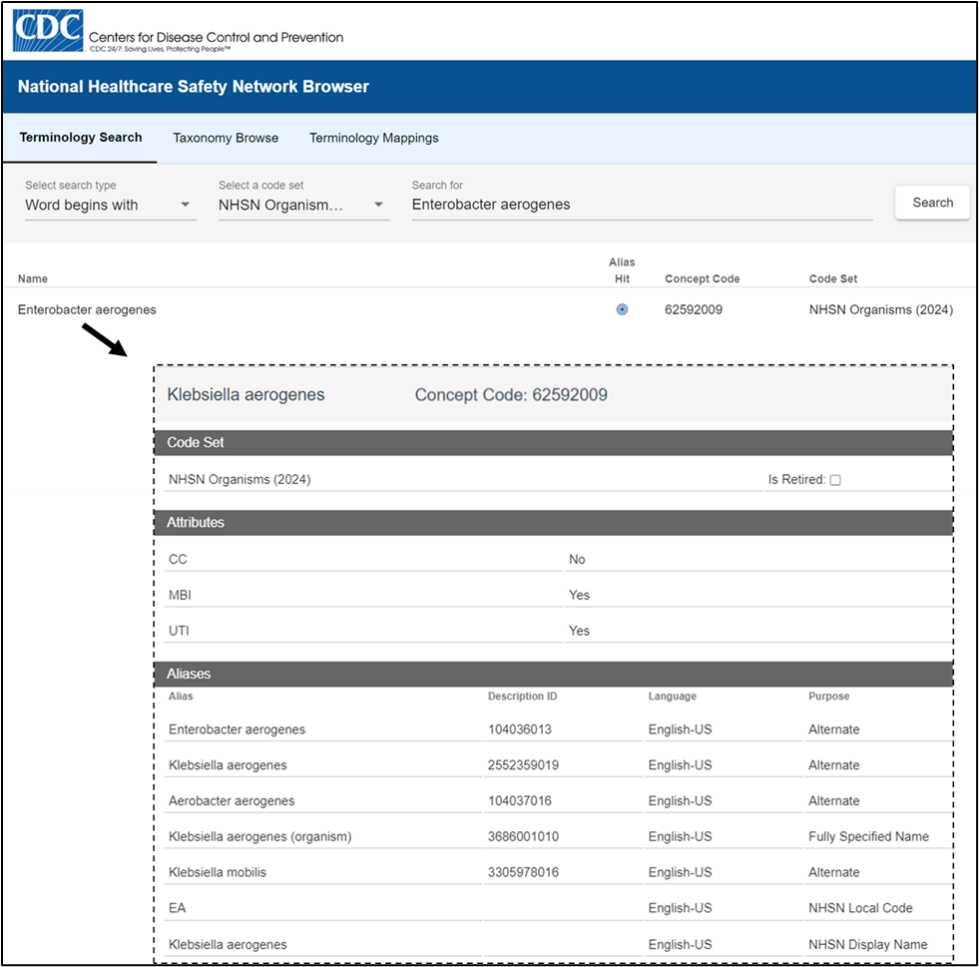
The National Healthcare Safety Network (NHSN) terminology browser for the Pathogen Codes SNOMED CT resource. CC: common commensals; MBI: mucosal barrier injury; UTI: urinary tract infection.

Identifying changes to SNOMED CT concepts and all associated NHSN-specific attributes in the *Pathogen Codes* resource is automated using the terminology software. Microorganism concepts that have moved to inactive status and updates to term descriptions due to changes in nomenclature are automatically flagged for review by subject matter experts from the protocols and data analysis teams with each new release of the code system. Maintenance is substantially more streamlined since the NHSN-specific metadata is now bound to SNOMED CT concept identifiers. Display names of microorganisms in dropdown lists in the web application are identical to the SNOMED CT Preferred Terms. All synonyms (ie, former and alternate names) for the microorganism concepts in the *Pathogen Codes* resource are automatically updated and are now searchable using a customized NHSN terminology browser (Figure 1). This simplified browser has eliminated the need to maintain a separate supplemental spreadsheet for users entering one event at a time into the web application. In addition to identifying synonyms, selected attributes that define reporting criteria for bloodstream infections and urinary tract infections (eg, the Common Commensals column in the *Pathogen Codes* asset) are displayed in the terminology browser for easy reference [9].

Since the NHSN codes are integral to analyzing data, change must be strategic to minimize disruptions among stakeholders. For maintenance purposes, existing NHSN mnemonic codes will persist as short alternate names (eg, EA for *Klebsiella aerogenes* that was formerly in the genus *Enterobacter*). Current analysis of electronically submitted data depends on translating standard codes from CDA files to NHSN codes. However, future-facing data aggregation and analysis will directly leverage the SNOMED CT ontology and non-semantic concept identifiers without the need for NHSN codes.

## Overcoming Challenges From Consequences of Program Expansion

Due to the considerable growth and success of the NHSN, legacy approaches to maintaining terminology assets were no longer sustainable. These challenges placed a demand on adopting a more automated solution to manage all terminology-related resources.

As a result of transitioning to the new terminology platform, reports can be configured to streamline export of customized assets derived from the master *Pathogen Codes* resource. This change will obviate the need to maintain separate companion spreadsheets for different use cases. In addition, the inherent version control and audit trail functionality will enable automated traceability.

**Figure 2.**
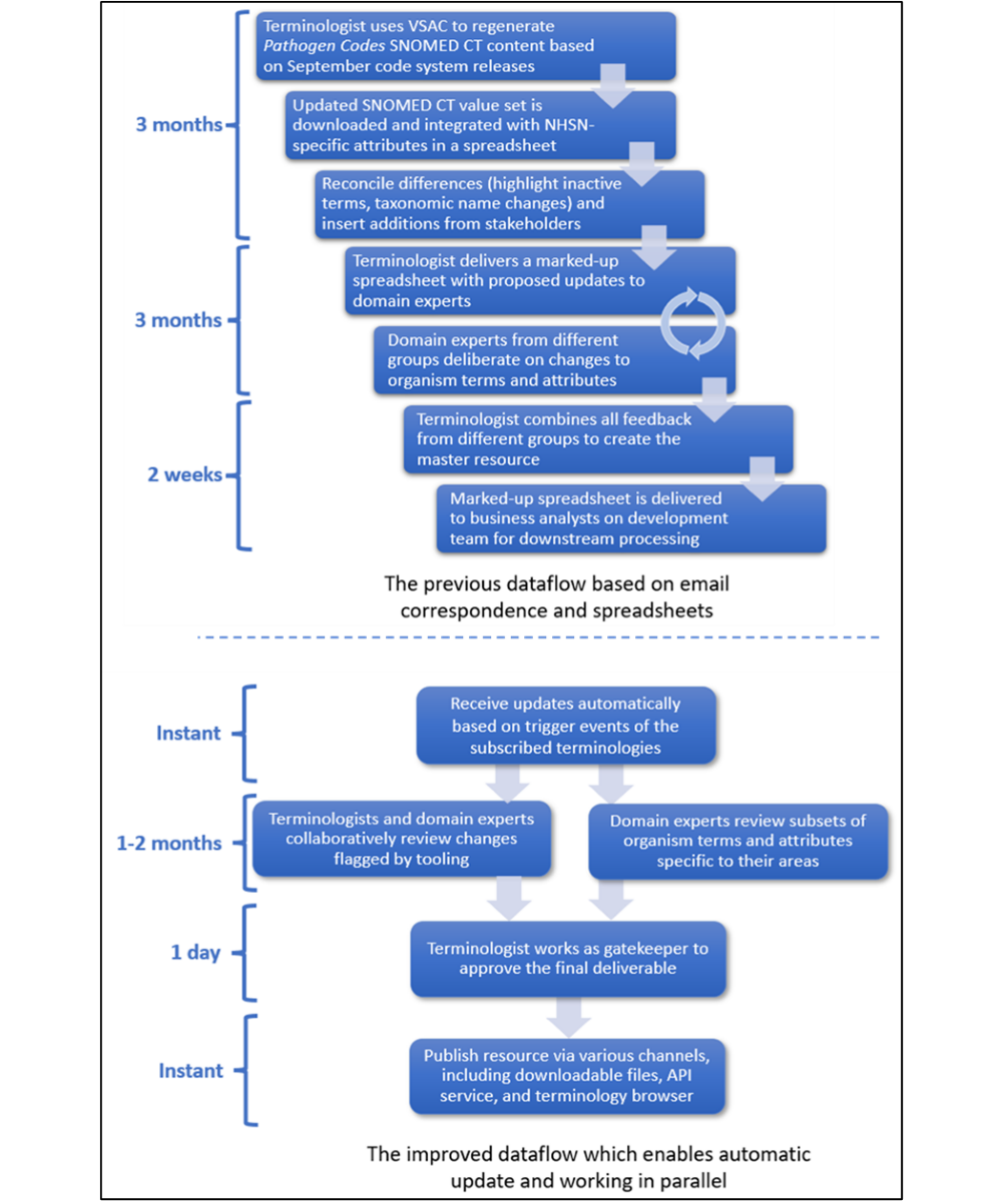
Streamlined data flow with the new approach.

Figure 2 illustrates the streamlined maintenance processes. Compared to the previous workflow, a major improvement is that reconciliation with new releases of SNOMED CT is essentially automated. After subscribing to a reference terminology in the platform, changes to NHSN vocabulary assets are applied seamlessly when triggered by new releases of the code system. Alleviating that burden of human intervention is amplified by features of the tooling that allow multiple domain specialists to work simultaneously on subsets of content related to their areas of expertise (eg, bloodstream infection or ventilator-associated events). Another feature that will standardize and streamline the maintenance process is the rich functionality for authoring rule-based value sets. This framework bridges the knowledge gap between domain experts and terminologists to apply rigorous inclusion and exclusion criteria for authoring and automatically refreshing value sets. Overall, the maintenance timeframe has been reduced from more than 6 months to less than 2 months.

In addition to an application that can automate and streamline the terminology maintenance procedures, the corresponding workflow design and education of the workforce are equally important. Enabling multiple collaborators to cohesively work in parallel on different aspects of a terminology asset not only fast tracks the overall processing time, but also reduces the risk of miscommunication. Meanwhile, training materials on essential terminology principles and methodologies should be offered to the workforce involved in defining requirements for terminology resources to transform routine processes. For example, a basic understanding of the approaches to building rule-based value sets is a fundamental step towards automating updates.

Preliminary progress with the NHSN terminology modernization efforts has been productive and promising. Our experience and lessons learned can be applied to similar use cases relying heavily on terminology resources that are subject to frequent updates. Further integration with other NHSN and CDC applications is a major future direction. A fully automated data pipe will be implemented based on the terminology services offered by the new platform, allowing systematic data provisioning to internal and external stakeholders. Meanwhile, all existing terminology resources will be migrated from the IDM to the new platform for improved maintenance and data sharing procedures. The NHSN is moving in the direction of maximizing electronic submission of data [10,11], but support for manual data entry in the web application and the current IDM will persist indefinitely to accommodate facilities with varying technical capacities and to support legacy surveillance reporting.

## Summary

In conclusion, the preparations and directions outlined have drastically eased the adoption and deployment of the new technical solution. Migration of the *Pathogen Codes* asset to the new terminology platform has been a proving ground for updating additional resources in the IDM containing standardized terminology content coupled to the NHSN codes. Being able to tether business rules for HAI reporting criteria to the SNOMED CT concepts of interest and repurposing NHSN codes as short, alternate-term descriptions are pivotal gains from implementing the new approach. Directly using the framework of terminology standards extended with NHSN-specific attributes will streamline maintenance tasks and keep the content more current. Although this use case is restricted to the SNOMED CT organism hierarchy for microorganisms identified in laboratory test results, benefits of this work will scale to other clinical, medications, and laboratory data elements critical for the next generation of NHSN reporting.

## Abbreviations

CDA: clinical document architecture
CDC: Centers for Disease Control and Prevention
HAI: healthcare-associated infections
IDM: information data model
NHSN: National Healthcare Safety Network
NNIS: National Nosocomial Infection Surveillance
SNOMED CT: Systematized Nomenclature of Medicine – Clinical Terms
SOP: standard operating procedure
TAM: terminology asset management
VSAC: value set authority center
